# Examining the relationship between sleep quality and depressive symptoms in Korean women engaged in soccer during the coronavirus pandemic

**DOI:** 10.3389/fpubh.2023.1282887

**Published:** 2023-11-17

**Authors:** Young-Jae Kim, Da-Som Lee, E-Sack Kim

**Affiliations:** Department of Physical Education of Chung-Ang University, Seoul, Republic of Korea

**Keywords:** COVID-19, depressive symptoms, Korean women, sleep quality, soccer

## Abstract

**Introduction:**

The COVID-19 pandemic has caused sudden changes to daily lives, such as self-isolation and social distancing, and has negatively affected sleep quality and patterns. The resulting psychological discomfort has caused many Korean women to experience depressive moods. Vigorous physical activity is considered effective in improving sleep quality and alleviating depressive symptoms. As a form of vigorous physical activity, soccer could be used to improve women's mental health. This study aimed to ascertain the effects of playing soccer on sleep quality and depressive symptoms in women.

**Methods:**

Non-face-to-face questionnaires were administered using Pittsburgh Sleep Quality Index to measure sleep quality and Patient Health Questionnaire-9 to measure depressive symptoms, targeting 200 of 297 soccer-playing Korean women aged 20–50 years, from October 13, 2022, to January 15, 2023. A total of 172 questionnaires administered to soccer participants were used, while 28 with insincere and double or no-responses were excluded. Additionally, 124 samples of non-exercise participants were collected, with the help of “EMBRAIN,” a Korean research and survey company. This study analyzed differences in sleep quality and depressive symptoms, and correlations and multiple regression analysis were performed.

**Results:**

The soccer group was shown to have a high quality of sleep. In relation to the effect of sleep quality on depressive symptoms, subjective sleep quality, sleep latency, sleep disturbance, use of sleeping pills, and daytime functional disorder had a significant effect. In the relation to the effect of sleep quality on depressive symptoms, significant effect was found in subjective sleep quality, sleep latency, sleep disturbance, and daytime functional disorder of soccer participants, and non-exercise participants displayed significant effect in subjective sleep quality, sleep disturbance, and the use of sleeping pills.

**Discussion:**

This study examined the effect of soccer participation on sleep quality and depressive symptoms among women. Soccer, which requires high activity and teamwork levels, improves sociability in women by enhancing their sense of belonging, self-confidence, and team spirit.

## 1 Introduction

The COVID-19 pandemic caused psychologically uncomfortable situations for many people, such as self-isolation and social distancing. Moreover, physical activity decreased globally, negatively affecting physical and mental health (1). Notably, spending too much time indoors long-term decreases the quality of life, due to anxiety, boredom, and harmful changes in lifestyle habits (2). According to the Korea Disease Control and Prevention Agency (3), since 2019, participation in moderate or vigorous physical activity has continuously decreased, and mental health (e.g., depression, stress) has also worsened.

Wilke et al. (4) reported that only 2 weeks of not participating in one's usual physical activities renders one more susceptible to depression. Craike et al. (5) said that a continued decrease in physical activity of females causes them to avoid participating in physical activities, and this participation reduces in the future. A decrease in physical activity can lead to changes in daily life that can increase symptoms of depression. This, in turn, has direct and indirect negative effects on sleep quality and patterns (6). According to McLean et al. (7), females experience more anxiety disorders, bulimia nervosa, and depressive disorders than males when infectious diseases are rampant, which has a negative influence on mental health. Especially, participation of females in physical activities can reduce stress and enhance wellbeing more than that of males, and such participation leads to a decrease in depression and anxiety (8). Therefore, it is essential to encourage women to participate in physical activities to improve their sleep quality and mental health, even in the post-COVID period.

Bueno-Notivol et al. (9) have found that post the COVID-19 pandemic, people, on average, feel depressed seven times higher than before. According to Jin and Kim (10), the decrease in physical activity following the emergence of COVID-19 has been a factor in increasing depressive moods among women and young adults (20–40 years old). COVID-19 can increase depression and negatively affect women's mental health through various mechanisms, including changes to daily life and society, loss of employment, reduced income, and restricted personal relationships (11). Low physical activity levels can reduce sleep quality via direct effects on depression and anxiety (12, 13). Moreover, individuals with severe anxiety and depression often experience sleep impairment, which includes waking up early in the morning, difficulty falling asleep, or frequent waking during the night (14).

People with high sleep responsiveness experience insomnia when they are highly stressed, whereas those with lower stress levels are less likely to experience insomnia. However, blockade due to COVID-19 increases the possibility of sleep disorder and insomnia because of stress (15). Na (16) reported that 6 out of 10 Korean women have experienced sleep problems since COVID-19, including waking up during sleep, difficulty falling asleep, and difficulty maintaining sleep. This causes mental stress, which, in severe cases, can lead to depression. According to the Sleep Review (17), the US has also experienced mental health issues due to COVID-19, with prescriptions of anxiolytics, antidepressants, and sleep medication increasing by approximately 30%. Thus, psychological discomfort due to COVID-19 causes women to suffer from impaired social function and self-determination, eating disorders, sleep disorders, lethargy, and physical changes (18).

Poor sleep also makes the recovery of full bodily functions difficult, negatively affecting one's health and daily life (19). Furthermore, poor sleep quality can lead to depression (20). Insomnia patients who face difficulty sleeping have an elevated risk of severe depression compared to healthy sleepers (21). Kakinami et al. (22) reported that the tendency for people to deny having sleep problems and not seeking treatment could lead to dependence on drugs or alcohol, further exacerbating sleep problems. The authors suggested vigorous physical activity to prevent this outcome. López-Bueno et al. (23) found that females, more than males, experience a more significant decrease in anxiety and an increase in emotional vitality when performing physical activities recommended by the WHO.

Meanwhile, the COVID-19 pandemic has caused a shift from participation in indoor physical activities to outdoors, to reduce contact with others. Shin (24) reported that outdoor sports, such as baseball, soccer, and golf, could have encouraged participation in physical activity during the COVID-19 pandemic because, compared to indoor sports, it is easier to adhere to disease control rules when participating in these sports. According to the 2021 National Sports Survey published by the Ministry of Culture, Sports, and Tourism (25), the sports with the highest participation rates prior to the COVID-19 pandemic were, in descending order, walking, mountain hiking, bodybuilding, and swimming. Following the pandemic, these were walking, mountain hiking, and soccer or futsal. Notably, as part of its COVID-19 control policies, the Korean government closed specific physical activity spaces, including indoor gyms; outdoor basketball, tennis, and badminton courts; and soccer fields. Nevertheless, the closure of only the soccer field closed was applied as an exception, increasing the participation rate in soccer (26).

In 2019, the female participation rate in soccer and futsal was extremely low; notably, the rate of women's registration in soccer and futsal clubs was 0.0%. In 2020, during the COVID-19 pandemic, this figure increased to 2.6%, and the percentage of women with experience participating in soccer or futsal also increased considerably, from 0.5% in 2019 to 15.6% (27, 28). Thus, while women's physical activity levels have been decreasing due to COVID-19, participation in soccer alone has been rising. In summary, women's engagement in sports has shifted from indirect participation, such as watching soccer, to direct participation in the sport. This is due to changes in leisure activity restrictions. These include the use of indoor gyms which were previously a common form of exercise among women (29). Additionally, the media promoted a positive image of women sweating as a result of vigorous exercise, which encouraged participation in soccer and other team sports that have previously been viewed as complex (30).

The “General Physical Activities Defined by Level of Intensity,” published by the US Centers for Disease Control and Prevention (31), defines soccer as vigorous physical activity and emphasizes the importance of outdoor activities. Soccer is a sport that requires high levels of cardiovascular endurance, and muscle and knee, ankle, and lower back strength, since participants are required to walk or run continually throughout the game (32). Friedrich and Mason (33) reported that soccer improved individual physical health and, as a team sport, increased social integration, a sense of achievement, and positive emotions, thus improving mental health. This reveals that playing soccer can have both positive physical and mental effects. Nevertheless, previous research has not elucidated the psychological effects of soccer participation among women during social difficulties such as the COVID-19 pandemic.

Therefore, this study analyzes depressive symptoms among the diverse psychological symptoms experienced during the COVID-19 and its correlation with sleep quality. Ultimately, the study results serve as a plan for women to live a healthy life by demonstrating that high-intensity physical activities such as soccer, can reduce psychological discomfort and enhance mental health even in post-COVID situations. Therefore, this study has the following hypotheses.

Hypothesis 1. There will be differences in sleep quality and depressive symptoms between soccer participants and non-exercise participants.
Hypothesis 2. Sleep quality will have a significant effect on depressive symptoms.
Hypothesis 3. Sleep quality of soccer participants will have a significant effect on depressive symptoms.
Hypothesis 4. Sleep quality of non-exercise participants will have a significant effect on depressive symptoms.

## 2 Materials and methods

### 2.1 Ethical considerations

To adhere to the ethical considerations with regard to the subjects, the researchers visited a female soccer circle in person and only distributed the questionnaires to the members, who agreed to join the research. Prior to this survey, screening and approval (1041078-202205-HR-128) by Ethics Committee of Chung-Ang University were obtained on the basis of “Helsinki Declaration” enacted in 1964.

### 2.2 Participants

This study administered a questionnaire to 296 Korean women aged 20–50 years during the COVID-19 pandemic. The questionnaire was administered to 200 soccer participants between October 13, 2022, and January 15, 2023. Of these, 28 questionnaires were excluded due to insincere, duplicate, or incomplete responses; thus, the responses of the remaining 172 soccer participants were included in the analysis. A sample of 124 exercise non-participants was also selected via the specialist research survey company, EMBRAIN. The sample was selected using non-stochastic convenience sampling, and the questionnaire was self-administered. The required sample size for this study was selected using Comrey and Lee (34) criteria (i.e., 100 = poor, 200 = adequate, 300 = good, 500 = very good, and 1,000 = outstanding).

### 2.3 Research instruments

#### 2.3.1 Demographic characteristics

The questions regarding demographic characteristics used in this study relate to age, type of occupation, and marital status. Questions about soccer activities include those of participants' soccer careers, participation hours, and the numbers of weekly participation. According to Frändin et al. (35), women show an increase in physical load and a decrease in sports participation and performance capability in daily life after 50 years of age. Soccer requires high physical activity; therefore, we included those above the age of 20 and below 50 in our study. Kim (36) specify that people exercising <3 times a week are at the preparation stage, and those exercising for more than 6 months are at the maintenance stage. The American College of Sports Medicine (37) classifies people doing sports activities for over 30 min at a time, 3 times a week or above as regular exercisers, this study developed the questions of soccer activities.

#### 2.3.2 Sleep quality

To investigate sleep quality in this study, the Pittsburgh Sleep Quality Index (PSQI) developed by Buysse et al. (38) was utilized. The PSQI is comprised of 19 questions across 7 subfactors: subjective sleep quality, sleep latency, sleep duration, sleep efficiency, sleep disturbance, use of sleep medication, and daytime dysfunction. A score of 0–3 points is calculated for each subfactor, based on corresponding responses, meaning the maximum total score was 21 points.

Scores closer to 0 indicated better sleep quality, and scores increased as sleep quality worsens. Sleep quality was classified using a cutoff score of 5 points, with those scoring ≤ 5 points classified as good sleepers and those scoring >5 points considered poor sleepers. The diagnostic sensitivity was 89.6%, and the specificity was 86.5% (38). When the reliability of the PSQI in this study was analyzed, a Cronbach's α of 0.644 was observed.

#### 2.3.3 Depressive symptoms

To measure the depressive symptoms state of soccer-playing women participating in this study, the Patient Health Questionnaire-9 (PHQ-9), which has been used in previous studies (39, 40) was utilized after adapting it for this study following a collaboration with a professor and a doctor in the social sciences of sports field. This scale is an instrument to diagnose depressive symptoms and is comprised of nine items that reflect the criteria for mental health diagnosis and major depressive disorder (41). Each item was scored from 0 (“Not at all”) to 3 (“Almost every day”). The maximum total PHQ-9 score was 27 points, and scores were classified as follows: minimal (0–4), mild (5–9), moderate (10–14), and severe (≥15). In a previous study, scores of ≥10 could indicate a depressive symptoms diagnosis (40). In this study, the average of nine questions was calculated and used. When we analyzed PHQ-9 reliability, a Cronbach's α value of 0.859 was observed.

### 2.4 Data analysis

All data underwent coding and data cleaning to achieve the study objectives. SPSS for Windows, version 25.0 (Microsoft Corp., Redmond, WA, USA), was used for data analysis. The specific analytical methods were as follows.

First, frequency analysis was performed to determine the demographic characteristics of the participants. Cross-tabulation analysis was then performed to investigate differences observed in the demographic characteristics, frequency of soccer participation, individual session duration, experience, sleep quality, and depressive symptoms depending on PSQI and PHQ-9 scores. To test the normality of the data, the skewness and kurtosis of the PSQI and PHQ-9 results were examined following West et al. (42) (i.e., skewness <3, kurtosis <8). The skewness ranged from 3.065 to 0.097, and the kurtosis ranged from 10.831 to −0.939, indicating that the hypothesis of normality was rejected. Notably, Pan et al. (43) and El Sayed et al. (44) have also used non-parametric statistics to investigate sleep quality, since the data failed to satisfy normality criteria.

Second, the reliability of the research instruments was tested by calculating Cronbach's α.

Third, since the data in this study did not fit the normal distribution criteria proposed by West et al. (42), the non-parametric Kruskal-Wallis test was used to investigate differences in sleep quality and depressive symptoms associated with soccer participation. Additionally, the Jonckheere-Terpstra test was used for *post hoc* comparisons with the Kruskal-Wallis H test, and the significance of all results was interpreted to a significance level of 0.05. When *n* > 30, the standard of central limit theorem is applied, and we can use parametric statistics even without normality. However, skewness (3.063~0.097) and kurtosis (10.831~-0.939) were both out of the standard even when the relaxed standard proposed by West et al. (42) was applied to this study. Furthermore, El Sayed et al. (44) targeted more participants than those of this study but produced results using non-parametric statistics.

Fourth, to investigate the effects of soccer participation on sleep quality and depressive symptoms, a non-parametric Spearman's correlation analysis was utilized. Although the study data was not normally distributed, Ramsey and Schafer (45) and Williams et al. (46) have stated that deviation from normality does not cause bias in regression coefficients or impair hypothesis testing. Since there are no problems associated with using regression analysis, we analyzed our data using multiple regression analysis.

## 3 Results

The participants' demographic characteristics were as follows. The most common age group was 20–29 years (144 persons, 48.6%), followed by 30–39 years (77 persons, 26.0%), and 40+ years (75 persons, 25.3%). The most common occupation was worker (151 persons, 51.0%), followed by college students (89 persons, 30.1%), and full-time homemakers (56 persons, 18.9%). There were 192 unmarried participants (64.9%) and 104 married participants (35.1%). The weekly frequency of participation in vigorous exercise was 2+ times per week for 108 participants (36.5%) and once per week for 64 participants (21.6%). The duration of each vigorous exercise session was 2+ h for 149 participants (50.3%) and <1.5 h for 23 participants (7.8%). Finally, the experience length of vigorous exercise was <2 years for 120 participants (40.5%) and 2+ years for 52 participants (17.6%).

In cross-tabulation analysis on the effects of soccer participation, significant differences were observed in age (χ^2^ = 58.419, *p* = 0.000), occupation (χ^2^ = 27.840, *p* = 0.000), marital status (χ^2^ = 16.443, *p* = 0.000), PSQI (χ^2^ = 10.746, *p* = 13.250), and PHQ-9 (χ^2^ = 13.250, *p* = 0.000) (Table 1).

**Table 1:** Participants' demographic characteristics (*n* = 296).

**Characteristics**		**Total, *n* (%)**	**Soccer participant**	**Exercise non-participant**	**χ^2^ (*p*)**
			**Frequency (%)**		
Age	20–29 years	144 (48.6)	96 (66.7)	48 (33.3)	58.419 (0.000)
	30–39 years	77 (26.0)	60 (77.9)	17 (22.1)	
	40+ years	75 (25.3)	16 (21.3)	59 (78.7)	
Occupation	College student	89 (30.1)	58 (65.2)	31 (34.8)	27.840 (0.000)
	Worker	151 (51.0)	99 (65.6)	52 (34.4)	
	Full-time homemaker	56 (18.9)	15 (26.8)	41 (73.2)	
Marital status	Unmarried	192 (64.9)	128 (66.7)	64 (33.3)	16.443 (0.000)
	Married	104 (35.1)	44 (42.3)	60 (57.7)	
PSQI	PSQI ≥5	143 (48.3)	97 (67.8)	46 (32.2)	10.746 (0.001)
	PSQI < 5	153 (51.7)	75 (49.0)	78 (51.0)	
PHQ-9	PHQ-9 (0–9)	264 (89.2)	163 (61.7)	101 (38.3)	13.250 (0.000)
	PHQ-9 (≥10)	32 (10.8)	9 (28.1)	23 (71.9)	
Weekly frequency of soccer participation	Once per week	64 (21.6)			
	Twice per week	108 (36.5)			
Duration of each soccer session	< 1.5 h	64 (7.8)			
	2+ h	108 (50.3)			
Experience in soccer participation	< 2 years	120 (40.5)			
	2+ years	52 (17.6)			

### 3.1 Differences in sleep quality and depressive symptoms depending on soccer participation

Table 2 shows the results of the non-parametric Kruskal-Wallis test to examine differences in sleep quality and depressive symptoms among women during the COVID-19 pandemic in association with soccer participation. Specifically, significant differences were observed in perceived sleep quality (χ^2^ = 16.816, *p* = 0.000), sleep latency (χ^2^ = 14.325, *p* = 0.000), sleep duration (χ^2^ = 11.210, *p* = 0.001), sleep disturbance (χ^2^ = 9.461, *p* = 0.002), daytime dysfunction (χ^2^ = 6.692, *p* = 010), and PHQ-9 (χ^2^ = 37.405, *p* = 0.000). The Jonckheere-Terpstra test was utilized to verify if there were differences between the groups, and differences were found in perceived sleep quality (*p* = 0.000), sleep latency (*p* = 0.000), sleep duration (*p* = 0.001), sleep disturbance (*p* = 0.002), daytime dysfunction (*p* = 0.010), and PHQ-9 (*p* = 0.000).

**Table 2:** Differences in sleep quality and depressive symptoms depending on soccer participation (*n* = 296).

		**Kruskal-Wallis test**				**Jonckheere-Terpstra test**	
**Dependent variable**	**Group**	***M*** **(** ***SD*** **)**	**Mean rank**	χ^2^	***p***	**Std. J-T**	***p*** **(two-tailed)**
Perceived sleep quality	Soccer participants	1.155 (0.725)	132.44	16.816	0.000	4.101	0.000
	Exercise non-participants		169.75				
Sleep latency	Soccer participants	1.341 (0.929)	133.45	14.325	0.000	3.785	0.000
	Exercise non-participants		168.35				
Sleep duration	Soccer participants	0.963 (0.892)	134.70	11.210	0.001	3.348	0.001
	Exercise non-participants		166.60				
Sleep efficiency	Soccer participants	0.294 (0.631)	145.44	0.723	0.395	0.850	0.395
	Exercise non-participants		151.59				
Sleep disturbance	Soccer participants	1.024 (0.431)	139.50	9.461	0.002	3.076	0.002
	Exercise non-participants		159.89				
Use of sleep medication	Soccer participants	0.159 (0.418)	146.10	0.567	0.451	0.753	0.451
	Exercise non-participants		150.65				
Daytime dysfunction	Soccer participants	1.280 (0.823)	137.84	6.692	0.010	2.587	0.010
	Exercise non-participants		162.20				
PHQ-9	Soccer participants	0.485 (0.445)	122.46	37.405	0.000	6.116	0.000
	Exercise non-participants		183.72				

### 3.2 Analysis of correlations between sleep quality and depressive symptoms in soccer participants

The result of Spearman correlation analysis—a non-parametric correlation analysis to understand the correlation between sleep quality and depressive symptoms in soccer participants—is presented in Table 3. As shown in Table 3: subjective sleep quality had no correlation with the use of sleeping pills; sleep latency had no correlation with sleep hours, the use of sleeping pills, and daytime functional disorder; sleep hours had no correlation with sleep latency, sleep efficiency, sleep disturbance, the use of sleeping pills, daytime functional disorder, and PHQ-9; sleep efficiency had no correlation with the use of sleeping pills, daytime functional disorder, and PHQ-9; sleep disturbance had no correlation with the use of sleeping pills and daytime functional disorder; and the use of sleeping pills had no correlation with PHQ-9. The other factors had significant positive correlation statistically. Additionally, no factors had a correlation coefficient over 0.80, which means there is no problem with multicollinearity.

**Table 3:** Analysis of correlation between sleep quality and depressive symptoms in soccer participants (*n* = 172).

	***M***	***SD***	**1**	**2**	**3**	**4**	**5**	**6**	**7**	**8**
1	1.006	0.587	1.000							
2	1.163	0.890	0.332^**^	1.000						
3	0.814	0.831	0.257^**^	0.101	1.000					
4	0.279	0.652	0.245^**^	0.207^**^	0.118	1.000				
5	0.959	0.437	0.371^**^	0.228^**^	0.143	0.198^**^	1.000			
6	0.134	0.358	0.100	0.097	0.013	0.038	0.093	1.000		
7	1.174	0.775	0.335^**^	0.129	0.182	0.069	0.152	0.417^**^	1.000	
8	0.359	0.371	0.376^**^	0.296^**^	0.146	0.124	0.304^**^	0.194	0.396^**^	1.000

^**^p < 0.01. 1, Perceived sleep quality; 2, Sleep latency; 3, Sleep duration; 4, Sleep efficiency; 5, Sleep disturbance; 6, Use of sleep medication; 7, Daytime dysfunction; 8, PHQ-9.

### 3.3 Analysis of correlations between sleep quality and depressive symptoms in exercise non-participants

The result of Spearman correlation analysis for understanding the correlation between sleep quality and depression in non-exercise participants is presented in Table 4. As shown: sleep latency had no correlation with sleep efficiency, sleep disturbance, and the use of sleeping pills; sleep hours had no correlation with sleep efficiency, sleep disturbance, the use of sleeping pills, daytime functional disorder, and PHQ-9; sleep efficiency had no correlation with sleep disturbance, the use of sleeping pills, daytime functional disorder, and PHQ-9; and sleep disturbance had no correlation with daytime functional disorder. The other factors showed significant positive correlation statistically. As there were no factors with a correlation coefficient of 0.80 or over, there is no problem with multicollinearity.

**Table 4:** Analysis of correlation between sleep quality and depressive symptoms in exercise non-participants (*n* = 124).

	***M***	***SD***	**1**	**2**	**3**	**4**	**5**	**6**	**7**	**8**
1	1.363	0.839	1.000							
2	1.589	0.928	0.522^**^	1.000						
3	1.169	0.935	0.271^**^	0.281^**^	1.000					
4	0.315	0.603	0.228	0.159	0.149	1.000				
5	1.113	0.407	0.345^**^	0.104	0.086	0.110	1.000			
6	0.194	0.489	0.259^**^	0.148	0.120	0.008	0.226	1.000		
7	1.427	0.866	0.587^**^	0.368^**^	0.143	0.064	0.097	0.332^**^	1.000	
8	0.659	0.479	0.595^**^	0.405^**^	0.133	0.014	0.384^**^	0.289^**^	0.396^**^	1.000

^**^p < 0.01. 1, Perceived sleep quality; 2, Sleep latency; 3, Sleep duration; 4, Sleep efficiency; 5, Sleep disturbance; 6, Use of sleep medication; 7, Daytime dysfunction; 8, PHQ-9.

### 3.4 Analysis of correlations between sleep quality and depressive symptoms

The result of partial correlation analysis with age as a covariate for analyzing the correlation between sleep quality and depression is indicated in Table 5. As shown here, the use of sleeping pills had no correlation with sleep efficiency, and there was no correlation between daytime functional disorder and sleep efficiency either. The other factors had significant positive correlation statistically. Additionally, there were no factors over 0.80 of correlation coefficient, indicating no multicollinearity problem.

**Table 5:** Analysis of correlation between sleep quality and depressive symptoms (*n* = 296).

	**Control variable**	**1**	**2**	**3**	**4**	**5**	**6**	**7**	**8**
1	Age	1.000							
2		0.458^**^	1.000						
3		0.291^**^	0.185^**^	1.000					
4		0.246^**^	0.220^**^	0.141	1.000				
5		0.370^**^	0.182^**^	0.123	0.160^**^	1.000			
6		0.209^**^	0.112	0.124	0.029	0.224^**^	1.000		
7		0.493^**^	0.267^**^	0.194^**^	0.056	0.169^**^	0.334^**^	1.000	
8		0.520^**^	0.366^**^	0.170^**^	0.125	0.402^**^	0.349^**^	0.443^**^	1.000

^**^p < 0.01. 1, Perceived sleep quality; 2, Sleep latency; 3, Sleep duration; 4, Sleep efficiency; 5, Sleep disturbance; 6, Use of sleep medication; 7, Daytime dysfunction; 8, PHQ-9.

### 3.5 Effects of sleep quality on depressive symptoms

Table 6 shows the results of multiple regression analyses used to investigate the effects of sleep quality on depressive symptoms. The variance inflation factor (VIF) was 1.089–1.824. Since this was smaller than 10, it indicates no multicollinearity problems. The Durbin-Watson coefficient was 1.939, which is close to the criterion value of 2; thus, it demonstrated the independence of the residuals. Significant effects were observed for perceived sleep quality (β = 0.249, *t* = 4.101), sleep latency (β = 0.156, *t* = 3.035), sleep disturbance (β = 0.225, *t* = 4.553), use of sleep medication (β = 0.168, *t* = 3.469), and daytime dysfunction (β = 0.185, *t* = 3.428). The explained variance of the final regression model was 40.5% (*R*^2^ = 0.405).

**Table 6:** Effects of sleep quality on depressive symptoms (*n* = 296).

**Dependent variable**	**Independent variable**	**Unstandardized coefficients**		**β**	***t***	**VIF**	**D-W**	***R* ^2^**	***F***
		**B**	**Standard error**						
PHQ-9	Constant	−0.179	0.060		−2.979^**^		1.939	0.405	29.727^***^
	Perceived sleep quality	0.153	0.037	0.249	4.101^***^	1.824			
	Sleep latency	0.075	0.025	0.156	3.035^**^	1.306			
	Sleep duration	−0.002	0.024	−0.004	−0.079	1.120			
	Sleep efficiency	−0.015	0.033	−0.022	−0.465	1.089			
	Sleep disturbance	0.232	0.051	0.225	4.553^***^	1.213			
	Use of sleep medication	0.179	0.052	0.168	3.469^***^	1.167			
	Daytime dysfunction	0.100	0.029	0.185	3.428^***^	1.438			

^**^p < 0.01, ^***^p < 0.001.

### 3.6 Effects of sleep quality on depressive symptoms depending on soccer participation

Table 7 shows the results of multiple regression analysis used to investigate the effects of sleep quality on depressive symptoms dependent on soccer participation, using a group as the selection variable. For the soccer participants, the VIF was 1.103–1.550. Since this was smaller than 10, multicollinearity was not a problem. The Durbin-Watson coefficient was 2.024, close to the criterion value of 2, and demonstrated the independence of the residuals. Significant effects were observed for perceived sleep quality (β = 0.167, *t* = 2.138), sleep latency (β = 0.145, *t* = 2.119), sleep disturbance (β = 0.217, *t* = 3.100), and daytime dysfunction (β = 0.245, *t* = 3.319). The explained variance of the final regression model was 35.7% (*R*^2^ = 0.357).

**Table 7:** Effects of sleep quality on depressive symptoms depending on soccer participation (*n* = 296).

**Selection variable**	**Dependent variable**	**Independent variables**	**Unstandardized coefficients**		**β**	***t***	**VIF**	**D-W**	***R* ^2^**	***F***
			**B**	**Standard error**						
Soccer participants (*n* = 172)	PHQ-9	Constant	−0.151	0.066		−2.284		2.024	0.357	12.993^***^
		Perceived sleep quality	0.105	0.049	0.167	2.138	1.550			
		Sleep latency	0.060	0.028	0.145	2.119	1.190			
		Sleep duration	−0.010	0.029	−0.022	−0.328	1.103			
		Sleep efficiency	0.033	0.038	0.057	0.862	1.119			
		Sleep disturbance	0.184	0.059	0.217	3.100^**^	1.246			
		Use of sleep medication	0.134	0.072	0.130	1.861	1.239			
		Daytime dysfunction	0.117	0.035	0.245	3.319^***^	1.394			
Exercise non-participants (*n* = 124)	PHQ-9	Constant	−0.065	0.123		−0.524		2.129	0.415	11.632^***^
		Perceived sleep quality	0.169	0.059	0.297	2.884^**^	2.091			
		Sleep latency	0.068	0.044	0.132	1.547	1.421			
		Sleep duration	−0.008	0.039	−0.016	−0.208	1.156			
		Sleep efficiency	−0.097	0.064	−0.112	−1.506	1.087			
		Sleep disturbance	0.243	0.093	0.204	2.615^**^	1.195			
		Use of sleep medication	0.201	0.075	0.206	2.684^**^	1.161			
		Daytime dysfunction	0.078	0.049	0.143	1.588	1.581			

^**^p < 0.01, ^***^p < 0.001.

For the exercise non-participants, the VIF was 1.087–2.091; since this was smaller than 10, again, multicollinearity was not a problem. The Durbin-Watson coefficient was 2.129, close to the criterion value of 2, thus demonstrating the independence of the residuals. Significant effects were observed for perceived sleep quality (β = 0.297, *t* = 2.884), sleep disturbance (β = 0.204, *t* = 2.615), and use of sleep medication (β = 0.206, *t* = 2.648). The explained variance of the final regression model was 41.5% (*R*^2^ = 0.415).

## 4 Discussion

This study investigated the relationship between sleep quality and depressive symptoms among Korean women during the COVID-19 pandemic, and how this was affected by playing soccer. The study findings are discussed below.

First, to verify Hypothesis 1, non-parametric statistics were used to analyze the differences in sleep quality and depressive symptoms between women that played soccer and those that did not. Significant differences were observed between the two groups in terms of perceived sleep quality, sleep latency, sleep duration, sleep efficiency, sleep disturbance, daytime dysfunction, and PHQ-9 values. Specifically, all sleep quality-related factors and PHQ-9 values revealed a higher mean rank for women who played soccer. This indicates that their overall sleep quality is better, and that negative internal factors, such as depressive symptoms, are lower among women that play soccer. Therefore, vigorous exercise, such as playing soccer, is associated with reduced depressive symptoms and improvements in everyday sleep quality, which has been confirmed by some researchers (47–49).

Elavsky and McAuley (47) analyzed sleep quality before and after walking (light physical activity) and practicing yoga (moderate physical activity), and found that light and moderate physical activity did not improve sleep quality. Jurado-Fasoli (48) analyzed changes in sleep quality following vigorous physical activity. They reported positive effects on overall sleep quality, total sleep duration, and efficiency. Meanwhile, Ironside et al. (49) reported that concerns such as infectious diseases, working from home, and childcare could cause a reduction in duration of hours slept among women. Nevertheless, women who participated regularly in vigorous aerobic exercise were shown to have better daily sleep quality than those who participated in light or moderate exercise, supporting the results of previous studies (48, 49). These similar demonstrations that vigorous exercise can improve sleep quality, further support our findings as well.

Myeong (50) previously reported that Korean women in their 20s and 30s had a severe lack of exercise, with lower rates of walking than among women in their 60s. Vuelvas-Olmos et al. (51) emphasized the importance of physical activity, reporting that a lack of sufficient physical activity during the COVID-19 pandemic could increase the risk of anxiety, depression, and stress due to persistent negative thoughts.

Nevertheless, there remains a severe lack of exercise spaces for women in Korea (52). Currently, due to the male-dominated exercise spaces and the lack of programs in which women can easily participate, it is difficult for them to engage in exercise (53). Additionally, the social environment makes it extremely difficult for women to exercise sufficiently. Even among individuals who manage to exercise, when personal circumstances prevent them from exercising, motivation can decline, leading to unhealthy stress (54). Blake et al. (55) and Bu and Chung (56) reported that social support from nearby friends and family, and environmental support that enables exercise participation could increase internal motivation for exercise. This suggests the importance of expanding facilities and developing programs to increase women's exercise participation rates, as well as strategies that promote continual participation. Exercise participation during a pandemic improves sleep quality, positively affects depressive symptoms as well as other forms of mental health, and provides physical and mental health benefits that can help individuals endure difficult circumstances.

Second, to verify Hypotheses 2– 4, the effects of sleep quality on depressive symptoms were examined, and significant effects of perceived sleep quality and sleep disturbance were observed among soccer participants and non-participants. Meanwhile, significant effects were associated with sleep latency, sleep disturbance, and daytime dysfunction among soccer players, as well as with using sleep medications among non-participants. Specifically, women who did not participate in regular exercise were more likely to use sleep medication to fall asleep.

Altevogt and Colten (57) reported that some people did not consider sleep problems important. Thus, they either did not recognize the need for a precise expert diagnosis, or they tended to avoid cognitive or behavioral treatments because of the perception of their immense potential financial burden. This can lead some people to develop dependencies on alcohol or sleep medication, which have negative physical and emotional effects and further exacerbate sleep problems (58, 59). Pacheco and Wright (60) found that women suffering from insomnia could be revitalized and could alleviate their depression by regularly participating in vigorous physical activity which subsequently improved their insomnia. The present study observed a significant effect from using sleep medication only among those who did not participate in exercise, indicating the need to improve one's natural environment through continual exercise rather than medication.

Kashefi et al. (61) examined sleep quality in 30- to 50-year-old women before and after engaging in moderate or vigorous aerobic exercise. Through exercise, participants' sleep durations became more consistent, sleep disruptions decreased, and they experienced positive changes in sleep latency and efficiency. Additionally, Ji et al. (62) also found that vigorous exercise had an even more significant effect on depressive symptoms and sleep quality compared with light exercise. Additionally, our study found that participation in soccer can positively affect sleep quality and depressive symptoms. In summary, increased participation in exercise has been associated with better sleep quality, including factors such as sleep latency and perceived sleep quality.

Yang (63) also reported the benefits associated with women playing soccer—a sport that has typically seen lower female participation rates due to its perception as a male sport. Specifically, soccer participation was found to positively affect personal health and increase the sense of friendship within groups. It also gave individuals a sense of pride and belonging, and helped them become self-confident, progressive women. Min et al. (64) examined people participating in vigorous group exercise (at an intensity sufficient to make participants' hearts beat faster and cause them to become out of breath). They reported that participants experienced greater happiness and stronger positive mental health effects than people exercising alone.

Yìǧìter (65) and Gothe et al. (66) reported that vigorous exercise improved psychological and physical self-esteem, increased pride in oneself, and ameliorated negative psychological states such as despair. Soccer—as a specific type of vigorous exercise—can increase self-esteem and positively affect mental health during a pandemic such as COVID-19. Therefore, in the future, it is essential to prepare measures that allow people to participate in sufficient physical activities, especially in future pandemic-like situations.

A study by Lemola et al. (67) found that people who slept 7–8 h showed lower self-esteem and less optimism to cope flexibly with certain situations compared with people who slept <6 h. Additionally, the authors reported that when lack of sleep persisted, it could lead to insomnia. Meanwhile, Swanson et al. (68) reported that women who could not sleep at least 7 h or who had irregular sleep patterns were at a considerably higher risk of depression and anxiety compared with those with normal sleep durations and patterns. Therefore, it is essential to relieve stress through appropriate moderate-to-vigorous physical activity. Participating in soccer or any other team-based sport that emphasizes teamwork over individual ability at a moderate-to-vigorous level can imbue participants with a sense of pride and belonging, increase friendships, promote sociability, and improve mental health, including self-esteem. If strategies are developed to increase the influx of women into team sports that have previously shown low female participation rates, it could improve sleep quality as well as mental and physical health of the participants.

This study has several limitations. First, only individuals participating in soccer were selected. In future studies, analyzing soccer players along with various other vigorous sports would be helpful in increasing female overall participation in sports and highlighting the positive aspects of physical activity. This would provide data to help improve sleep quality and mental health through participation in sports while adapting to the needs of individuals. Second, as this was a cross-sectional study, there are limitations in considering individuals' social and psychological circumstances when they completed the questionnaire. Future studies should use a longitudinal design, such as repeated observations or pre-post comparisons, which would provide more detailed information about the relationships between sleep quality in women in association with exercise and depressive symptoms. This would also help promote exercise participation during disasters such as the COVID-19 pandemic. Third, this study examined sleep quality and depression without considering demographic characteristics, such as occupations, age, and marriage status of soccer participants and non-exercise participants. If further research considers controlled situations exactly, more specific findings will come out. Fourth, this study lacks objective psychopathological evaluations and has a sample bias of research participants collected online. Moreover, the soccer participants were mostly 20–30 years old, whereas most of non-exercise participants were 40–50 years old. Therefore, further research will need to use tools for objective evaluations and target participants of similar age under controlled situations to study depression and sleep quality of females participating in sports more closely.

## 5 Conclusion

The COVID-19 pandemic has had negative effects on sleep quality and depressive symptoms in women. These result from reduced physical activity due to, for example, difficulty participating in indoor activities. Consequently, outdoor activities were recommended and female participation in soccer increased. Therefore, we investigated the relationships between female soccer participation during the COVID-19 pandemic and sleep quality and depressive symptoms. Our conclusions are as follows.

First, when we analyzed differences in the measured variables depending on soccer participation. We observed significant differences in perceived sleep quality, sleep latency, sleep duration, sleep efficiency, sleep disturbance, daytime dysfunction, and PHQ-9 values. Particularly, all sleep quality sub-factors showed significant differences, and the PHQ-9 revealed higher mean ranks in women who played soccer. Since there exists a lack of space for women to exercise and participate in vigorous sports activities, it is essential to construct adequate such spaces. We propose that expanding exercise spaces would be one way to overcome the challenging environmental circumstances associated with COVID-19, which will improve sleep quality and positively affect mental health, including depressive symptoms.

Second, when examining the effects of sleep quality on depressive symptoms, perceived sleep quality and sleep disturbance were found to have significantly affected both soccer participants and non-participants. Meanwhile, sleep latency, sleep disturbance, and daytime dysfunction had significant effects on soccer participants, while the use of sleep medication significantly affected non-participants. Suitable moderate-to-vigorous exercises can increase a sense of belonging and pride, enhance friendships, and improve sociability, while positively affecting mental health, including self-esteem.

Therefore, exercise such as soccer can make participants more sociable by enhancing a sense of belonging and improving the self-confidence of exercise groups. Furthermore, continued efforts to encourage non-exercise participants to engage in exercise can increase their self-esteem and improve their mental and physical health.

## Data availability statement

The raw data supporting the conclusions of this article will be made available by the authors, without undue reservation.

## Ethics statement

The studies involving humans were approved by Chung-Ang University Institutional Review Board (1041078-202205-HR-128). The studies were conducted in accordance with the local legislation and institutional requirements. The participants provided their written informed consent to participate in this study. Written informed consent was not obtained from the individual(s) for the publication of any potentially identifiable images or data included in this article because in the case of soccer participants, since a face-to-face survey was conducted, it was considered that they agreed to participate in the study only by participating in the survey.

## Author contributions

Y-JK: Methodology, Validation, Writing—review & editing. D-SL: Investigation. E-SK: Writing—review & editing, Conceptualization, Formal analysis, Methodology, Validation, Writing—original draft, Writing—review & editing.
